# Dietary survey of poultry egg intake among residents in Kunming city, southwest China

**DOI:** 10.3389/fnut.2024.1314924

**Published:** 2024-03-06

**Authors:** Rui Wang, Yugao Wu, Chenxi Zhang, Chengyao Liang, Xiran Xia, Ximan Gao, Jing Fang

**Affiliations:** ^1^Institute for Health Sciences, Kunming Medical University, Kunming, China; ^2^Collaborative Innovation Center for West Ecological Safety (CIWES), Lanzhou University, Lanzhou, China

**Keywords:** dietary survey, poultry egg, food intake, consumer preference, dietary habit

## Abstract

**Background:**

For almost all people, eggs can be a wholesome addition to the diet. However, there is insufficient applicable data to evaluate the poultry egg intake of people in the city of Kunming located in southwest China.

**Objectives:**

To understand the situation of egg consumption among local residents in Kunming via a dietary survey.

**Methods:**

Residents living in three places of Kunming were chosen using a multi-stage random sampling method. The recall methods of 3-day food intake and 1-month food intake frequency were used to assess the quantity and frequency of poultry egg dietary intake of local people.

**Results:**

Of the 1,118 respondents, 565 (50.54%) were female and 553 (49.46%) were male with age range 0.5–91 years old. Egg consumption was widespread among the survey respondents with 88.01% reporting hen egg ingestion, but the dietary intake of other poultry eggs such as duck, quail, and goose eggs were much less frequent. The medium daily intake of hen eggs was 20.00 g/d with greater amount of hen egg consumption in older age groups. However, when calculated on a body-weight basis, the median amount of hen eggs consumed daily per kilogram of body weight for all survey respondents was 0.47 g/kg/d whereas this indicator for children was 1.33 g/kg/d, becoming the greatest among all age groups.

**Conclusions:**

Our study obtained a better understanding of poultry egg intake among residents in Kunming city and calculated the egg intake kilogram of body weight that can be a useful reference to inform the development of more accurate dietary recommendation. These results also provide basic data for nutrition monitoring and dietary exposure risk assessment of poultry egg intake.

## 1 Introduction

Rich in high-quality protein, vitamins, and other bioactive components, eggs are regarded as a very healthy food (1, 2). Eggs are affordable, have a wide variety of culinary uses and a moderate calorie content, making them accessible to most people (3). Eggs can be a nutrient-dense addition to the diet for individuals at various stages of life because they are also relatively rich in fat-soluble substance (4). Specifically, eggs could be especially helpful in the diets of elderly, the pregnant, and children who are at risk of having inadequate nutrient intakes (5).

Egg production has become a major agricultural sector and an integral part of global food supply due to the growth of the world population (6). The demand for eggs worldwide is still rising quickly. Egg consumption has climbed by roughly 40% during the previous 20 years (7). Following its rapid economic development, Chinese dietary patterns have been drastically changing, moving away from traditional diets (mainly cereals and starchy roots, with few animal sources) to diets high in fat, particularly saturated fat, and low in carbohydrates, and containing more energy-dense foods (8–11). Due to an increase in meat consumption in China since 2002, Chinese Dietary Guidelines have begun recommending appropriate consumption of poultry, eggs, fish and lean meat for urban and rural populations as well as reducing consumption of fatty meat and animal oil for those living in large cities (12, 13). However, data on Chinese people's intake of poultry eggs was relatively limited compared with meat consumption (14–16).

Nowadays, China produces and consumes more than 40% of egg in the world with produced egg reaching 33 Mt in 2019 (7). The China Health and Nutrition Survey reports that the consumption of eggs among Chinese residents increased continuously from 2002 to 2010–2012 and the average daily egg intake of Chinese residents in 2010–2012 reaching 24.3 g (17). However, due to different economic development levels, the intake of eggs among Chinese residents exists regional disparities.

Yunnan is a frontier and less-developed province in China whose capital is Kunming city. The whole province is located in a mountainous and plateau region with an altitude of 1,891 m in downtown area of Kunming city. To our knowledge, there have been no studies on egg intake of residents in this city. In order to obtain a better understanding of egg consumption among residents in this plateau city, we conducted an egg-consumption dietary survey among local residents in Kunming. The results of this survey may provide useful reference to inform the development of more accurate dietary guidelines in similar settings.

## 2 Materials and methods

### 2.1 Study design and population

We adopted a design of retrospective dietary survey in this research. The respondents were selected by using multi-stage random sampling method. There are 7 districts, 6 counties and 1 county-level city in Kunming city. Three places in Kunming, namely Wuhua district, Anning city and Luquan county were selected representing high, middle and low economic developmental areas in the city respectively. The probability proportional sampling method is adopted to select sample streets from each of the three-sampled places and to determine the population to be investigated in each street.

Residents who have been living in the selected street area for more than 6 months and could cooperate with investigators were eligible for inclusion in the study. Residents, who could not accurately recall the dietary of poultry eggs in the past 3 days and 1 month, were excluded.

### 2.2 Survey implementation and data collection

We used a structured-questionnaire to collect poultry egg dietary intake information by face-to-face interviews of respondents. Before each interview, written consent was obtained from the respondent. The questionnaire contained 4 sections. The first section included socio-demographic basic information (age, gender, height, weight, etc). The second and the third sections of the questionnaire gauged the dietary intake quantity and frequency of poultry eggs by using 3-day food records over 24-hour recall method and 1-month food frequency method (18). In the last section of the questionnaire, we investigated respondent's preference for ways of egg cooking and eating time. We obtained information about egg intake of children under 3 years old by asking parents or caregivers.

The survey was conducted from August 1st to October 5th, 2020. In total, 1,118 residents were surveyed in the pre-determined streets. Before the formal survey, in July 2020, a pre-test of the questionnaire was conducted to test its feasibility. In both the pre-survey and formal survey, the data were collected by pre-trained interviewers who are undergraduates and postgraduate students of public health in Kunming Medical University.

### 2.3 Data management and statistical analysis

EpiData version 3.1 was used to enter the survey data. Double-entry was applied to ensure input quality. Statistical analyses were performed using R software (version 4.1.1). Data of non-normally distributed continuous variable are presented as the median and interquartile range. Categorical variables are presented as frequencies (n) and percentages (%). Chi-square test was used to analyze demographic data and the Rank-sum test was used to compare the differences between studied variables in different age groups and respondent's preference for poultry egg foods. Values of *p* < 0.05 were considered statistically significant.

## 3 Results

### 3.1 Age, gender, and physical characteristics of the surveyed respondents

**Figure 1:**
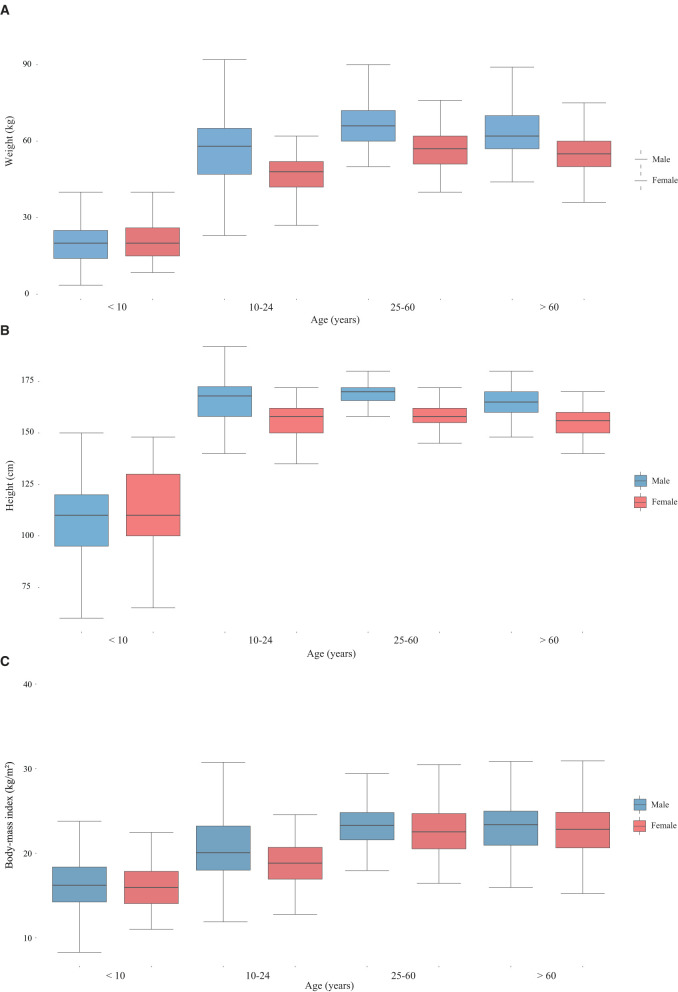
The weight **(A)**, height **(B)** and BMI **(C)** distribution of surveyed respondents (by gender).

A total of 1,118 residents were included in this analysis. Among them, 553 (49.46%) were male and 565 (50.54%) were female. Of those surveyed, 7 were pregnant and 35 were lactating women. The age range of all surveyed respondents was 0.5–91 years old with the median age 29 (P_25_: 10, P_75_: 60) years old. We divided those surveyed respondents into 4 age groups: child group (< 10 years old), adolescent group (10–24 years old), adult group (25–60 years old) and elder group (>60 years old). The age distribution of the male and female groups did not differ statistically significant (χ^2^ = 7.537, *p* = 0.057) (see Table 1). Figure 1 shows the distribution of body mass index (BMI), height, and weight of survey respondents.

**Table 1:** Gender and age distribution of surveyed respondents.

**Age (years)**	**Male**	**Female**	**Total**
< 10	135 (24.41)	125 (22.12)	260 (23.26)
10–24	139 (25.14)	121 (21.42)	260 (23.26)
25–60	140 (25.32)	184 (32.57)	324 (28.98)
>60	139 (25.14)	135 (23.89)	274 (24.51)
Total	553 (100.00)	565 (100.00)	1,118 (100.00)

### 3.2 Intake frequency of poultry eggs over the last 1 month prior to the survey

**Figure 2:**
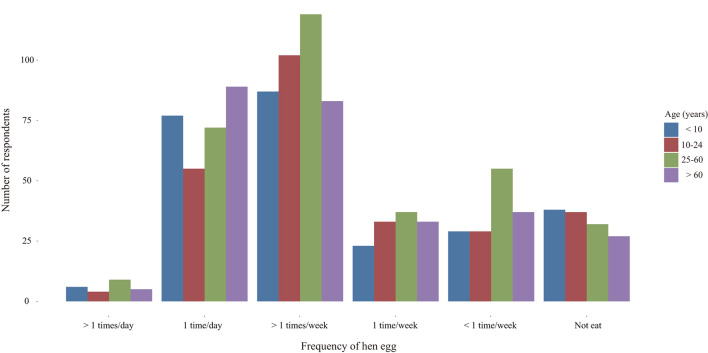
Intake frequency of hen egg in different age groups.

In order to obtain the information of intake frequency of poultry eggs, we investigated the dietary intake of respondents in the past 1 month. Egg consumption was widespread among the survey respondents with 88.01% reporting hen egg ingestion (Figure 2). Among those who eat eggs, 26.21% ingested hen eggs once a day (see Supplementary material). There were no significant differences in the intake frequency of hen egg between different genders (*z* =-0.289, *p* = 0.773) and age groups (*H* = 5.187, *p* = 0.159).

**Figure 3:**
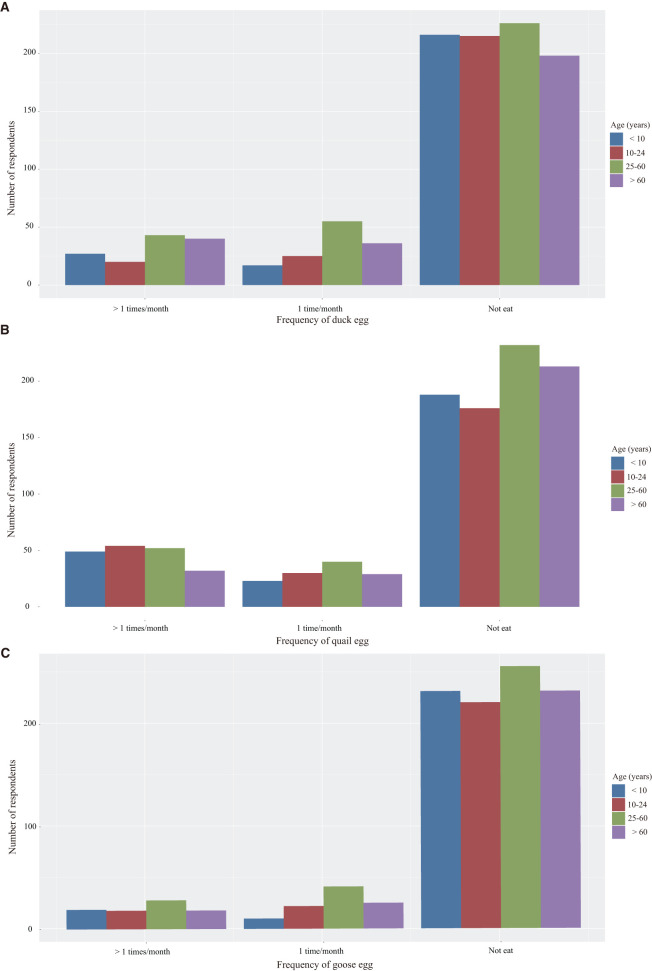
Intake frequency of duck **(A)**, quail **(B)** and goose **(C)** eggs in different age groups.

As shown in Figure 3, most of the respondents did not ingest duck (76.48%), quail (72.36%) and goose eggs (83.81%) in the past 1 month. There were 3.04%, 5.37% and 1.25% of respondents who ingested duck, quail and goose eggs 5 times or more per month. There were no significant differences in the intake frequency of duck, quail and goose egg between the males and females (*z* = −1.473, *p* = 0.141; *z* = −1.426, *p* = 0.154; *z* = −1.329, *p* = 0.184), however, the differences of intake frequency of duck, quail and goose egg among different age groups were statistically significant (*H* = 21.972, *p* < 0.001; *H* = 8.254, *p* = 0.041; *H* = 9.721, *p* = 0.021). Compared with child group and adolescent group, duck eggs were consumed more frequently in adult group and elder group. The intake frequency of quail egg in adolescent group was higher than that in elder group whereas the intake frequency of goose egg in adult group was higher than that in child group.

### 3.3 Intake of poultry eggs over the last 3 days prior to the survey

We collected the dietary intake of poultry eggs of survey respondents over the last 3 days prior to the survey and calculated the intake per day (g/d) and the intake per kilogram body weight per day (g/kg/d). Except for hen eggs, the median daily intake of duck, quail and goose eggs were all 0 g/d. As shown in the Table 2. The average intake of hen eggs was 34.68 g/d for men and 36.19 g/d for women. Among pregnant and lactating women, the average intake of hen eggs was 71.71 g/d. There were no significant differences in the daily intake of hen eggs (per day and per kilogram body weight per day) between males and females (*z* = −0.684, *p* = 0.494; *z* = −1.036, *p* = 0.300), but there were statistically significant differences in the intake of hen eggs (per day and per kilogram body weight per day) among different age groups (*H* = 20.905, *p* < 0.001; *H* = 64.929, *p* < 0.001). The intake per day of adolescent group was lower than that in other age groups, and the intake per kilogram body weight per day in adolescent group was also lower than that in elder group. Moreover, the intake per kilogram body weight per day in child group was greatest among all age groups. For the intake of duck, quail and goose eggs, see the Supplementary material.

**Table 2:** Dietary intake of hen eggs per day (g/d) and kilogram body weight per day (g/kg/d).

**Group**	**Age (years)**	**Per day**				**Per kilogram of body weight per day**			
		**Mean**	**P**_25_	**P**_50_	**P**_75_	**Mean**	**P**_25_	**P**_50_	**P**_75_
Male		34.68	0.00	20.00	60.00	0.98	0.00	0.40	1.10
	< 10	38.26	0.00	20.00	60.00	2.28	0.00	1.48	3.00
	10–24	28.15	0.00	20.00	40.00	0.57	0.00	0.27	0.81
	25–60	31.38	0.00	20.00	40.00	0.47	0.00	0.29	0.67
	>60	41.04	0.00	40.00	60.00	0.66	0.00	0.57	0.97
Female		36.19	0.00	20.00	60.00	0.95	0.00	0.50	1.16
	< 10	33.78	0.00	20.00	60.00	1.86	0.00	1.05	3.17
	10–24	22.65	0.00	20.00	40.00	0.53	0.00	0.38	0.82
	25–60	44.20	0.00	34.42	60.00	0.78	0.00	0.59	1.07
	>60	39.64	0.00	30.00	60.00	0.70	0.00	0.57	1.07
Pregnant and lactating women		71.71	25.00	60.00	102.75	1.30	0.38	1.01	1.84
Total		35.44	0.00	20.00	60.00	0.96	0.00	0.47	1.14
	< 10	36.10	0.00	20.00	60.00	2.08	0.00	1.33	3.05
	10–24	25.59	0.00	20.00	40.00	0.55	0.00	0.31	0.82
	25–60	38.66	0.00	20.00	60.00	0.65	0.00	0.39	0.92
	>60	40.35	0.00	40.00	60.00	0.68	0.00	0.57	1.00

### 3.4 Respondent's preference for poultry eggs

**Figure 4:**
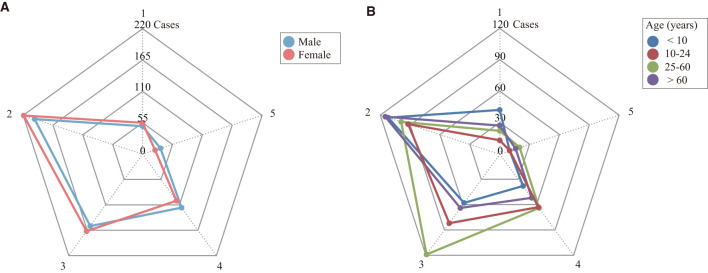
Respondent's preference for poultry eggs in different genders **(A)** and age groups **(B)**.

We collected data on respondent's preference for poultry eggs. The options of this question are “very like”, “like”, “moderate”, “dislike” and “very dislike”, ranging from 1 to 5. Among all the surveyed respondents, the proportion of “very like” to “very dislike” is 9.30%, 37.39%, 28.89%, 19.41% and 5.01%, respectively. There were no statistically significant differences in the distribution of poultry eggs preference between male and female (*z* = −1.725, *p* = 0.084), but the differences in the distribution of poultry eggs preference among different age groups were statistically significant (*H* = 31.741, *p* < 0.001). Compared with children, more adolescents and adults dislike eating poultry eggs. As shown in Figure 4.

Table 3 presents the timing at which respondents usually eat eggs. Among them, 29.07% of respondents would choose to eat an egg in the morning while 10.20% and 5.55% would prefer eating egg in the afternoon and evening. Although some respondents had a specific time for eating eggs, more than half of respondents (55.19%) had no set schedule and would have eggs whenever they were available. The most common ways of cooking eggs are presented in Table 3. It shows that 41.32% respondents preferred hard-boiled egg while the others like eggs cooked in other ways including fried (6.26%) and stewed (1.34%).

**Table 3:** Respondent's preference for ways of egg cooking and eating time.

**Variable**	***N* (%)**
**Timing for eating eggs (*****n*** = **1,118)**	
Morning	325 (29.07)
Afternoon	114 (10.20)
Evening	62 (5.55)
Any time of day	617 (55.19)
**Favorite ways of cooking eggs (*****n*** = **1,118)**	
Boiled	462 (41.32)
Fried	70 (6.26)
Steamed	15 (1.34)
Any other cooking ways	571 (51.07)

## 4 Discussion

To our knowledge, this is the first survey to investigate the food consumption of poultry eggs among residents in Kunming city, Yunnan province of China, a plateau area and also part of the upstream of Mekong region. This is also the first study that investigated the consumption of various poultry eggs in this city.

### 4.1 The level of egg dietary consumption

According to the World Agricultural Supply and Demand Estimates Report (19), the U.S. consumed 286.5 eggs per capita (number) in 2020. The 2020–2025 U.S. Dietary Guidelines advise egg as part of a healthy dietary pattern and provide recommendation for egg intake by life stage (20). The Chinese Dietary Guidelines (CDG) 2022 suggested intake 50g/d egg for adults, 25–40 g/d egg for children aged 6–10 years and 40–50 g/d egg for children aged 11–13 years (21). Our study found that the median daily intake of hen eggs among residents in Kunming was 20 g/d, indicating more than half of the surveyed population did not reach the recommended amount of CDG. A study published in 2011 in Jiangsu province, a better off province in China, analyzed the association between egg consumption and diabetes mellitus showing the average intake of eggs was 35.1 g/d in males and 30.7 g/d in females (22), similar to the amount consumed by the population in our survey (34.68 g/d for the male and 36.19 g/d for the female). A case-control study in Guangdong province, another better off province in China, in 2008 found that the median daily egg intake in breast cancer cases was 14.28 g/d and the control group was 16.02 g/d (23), smaller than the amount found in our survey. However, the times of the two above-mentioned surveys were more than 10 years ago, making them less comparable to our survey. In the past decade, the intake of egg by Chinese residents has been slightly increasing. A survey on Chinese residents' diet and chronic diseases reported that daily egg consumption of Chinese people grew from 23.7 g in 2002 to 24.3 g in 2012 (24). However, even compared with the data in 2012, the median intake of egg among local people of Kunming was still at a lower level. Our survey found a considerable number of adults (53.31%) dislike eating eggs or have a dispensable attitude toward eating egg. This may be the reason for the low per capita egg intake of participants in our survey.

### 4.2 Food preference, traditional believe, and avian influenza might affect egg intake

The traditional Chinese diet consists primarily of grains and vegetables, with very few animal products (25). Since the implementation of economic reform and opening policy in China in 1978, tremendous changes have occurred in Chinese society, including in the diet of Chinese people (26). In addition, over the past few decades China has taken significant steps toward improving nutrition, including advised increasing consumption of egg, fruit, and dairy products, however, egg intake of Chinese people had only increasing 1 g during a 10 year period (24), which is much smaller compared with the increasing of pork consumption in China (25). Our survey in Kunming city again found out that egg consumption of local residents remained smaller, far below the CDG recommendation. Compared with pork, poultry egg is more affordable and easier to cook, but its consumption did not increase as it should have been. The reasons behind this phenomenon need further study. However, three factors might contribute to this situation. First is food preference, our survey showed that 53.31% adults dislike eating eggs or have a dispensable attitude toward eating egg, which may partially explain the lower egg consumption among survey respondents. Second is the impact of traditional believe. Traditional Chinese Medicine has the symmetrical concept of cold and hot, similar to the concept of “Yin” and “Yang.” This concept has been extended to the domain of food and all food items can be divided according to their cold/hot qualities, this believe has guided the development of perceived healthful eating habits in Chinese cultures for thousands of years (27). Duck egg is considered a cold food item while chicken egg is considered a mild food; therefor duck eggs are usually not provided by families to children as shown by our survey. Third is the impact of avian influenza. Since 2004, episodes of avian influenza have been reported in East Asia, Southeast Asia, and Europe. Five waves of avian influenza occurred in mainland China from 2013 to 2017 (28). Consequently, egg production and consumption were adversely affected. Other factors that affect household purchase and consumption of eggs include the quality and safety of egg products, the nutritional value, the appearance, the price, and the brand (29).

### 4.3 Implications for egg intake evaluation and dietary recommendation

Because of their comparatively high dietary cholesterol level, eggs are a contentious food item (4). Some studies have identified eggs as element of a “healthy” or “prudent” eating pattern (30, 31). Egg consumption reduces the risk of mortality in adults by 17%, according to a study conducted in China in 2022 (32). In another study published in 2022, moderate-to-high consumption of eggs was found to reduce the risk of cardiometabolic factors among Chinese adults (33). Others have found eggs as components of dietary patterns linked to an increased risk of unfavorable outcomes such as overweight and obesity, metabolic syndrome, and insulin resistance (34, 35). Usual protein intake was measured using grams per day, grams per kilogram ideal body weight, and percentage of calories basis. The estimated average requirement (EAR) for protein is based on grams per kilogram of body weight (36). Furthermore, for some diseases with special requirements for protein intake, such as cardiovascular diseases, renal insufficiency and chronic hepatic disorder (37–39), the recommended protein intake is usually presented in grams per kilogram body weight per day. However, poultry egg as one of the important animal protein sources for human, there was no research on the egg food intake per kilogram of body weight and this indicator was neither recommended in Dietary Guidelines nor reported in the national food and nutrition surveillance of various countries. However, our study revealed that the daily intake of eggs in child group is not greater compared with other age groups, but after divided by individual body weight, the egg intake per kilogram body weight per day in child group is the greatest among all age groups. Thus, if we only investigate food intake without considering individual weight, it may cause misestimation of the actual intake of egg by different age groups. This also indicates that dietary recommendation of egg intake should be improved from xx g/day to xx g/body weight/day. In addition, current recommendations for dietary protein intake is 0.8 g/kg/d for all individuals, regardless of age or gender, this one-size-fits-all protein recommendation does not consider age-related changes in metabolism, immunity, hormone levels, or progressing frailty (40), therefore more accurate dietary guidelines are needed. Our study calculated the egg intake per kilogram of body weight by different age and sex groups that can be a useful reference to inform the development of more accurate dietary recommendations in the future.

### 4.4 Implication for exposure assessment of harmful chemicals via egg intake

Children's intake of eggs per kilogram of body weight per day was greatest among all age groups in our survey, which also means that children may be exposed to higher harmful contaminants contained in egg. A comparable research used data from the National Health and Nutrition Survey (USA) 2001–2002 to calculate average protein intake in grams per kilogram body weight showed that young children consume the most protein (4.4 ± 0.10 g/kg) whereas older women consume the least (1.0 ± 0.02 g/kg) (36). In many risk assessments, especially those involving chemical exposure, where risk is frequently represented as a function of the dosage of chemicals received per unit body weight, average body weight has traditionally been a crucial input parameter (41). For the purpose of evaluating dietary risks, accurate values of body weight at each age are required. According to a research, children are exposed to various environmental chemicals at excessively high levels and children consume more food per pound of body weight than adults (42). These results mean that children will be exposed to environmental pollutants in food and water at higher rates than adults. Our survey provided basic data for the risk assessment of contaminant exposure via poultry egg intake in Kunming city.

### 4.5 Limitations

The retrospective dietary survey method was used in this study and thus recall bias would impair data accuracy to certain extent. In addition, the dietary patterns of the respondents might change with the seasons that is related to cyclical available of food, which may have implication for survey results. Our study was conducted in late summer and early autumn and therefore, the survey results may only indicate the egg intake of the respondents in that season.

## 5 Conclusions

Our survey results showed that nearly 90% of the survey respondents in Kunming consumed egg. Most of the residents ingested hen eggs at least once a week. Children ingested more eggs on a body-weight basis than other age groups. Boiled eggs were the most popular food item in the research area. The results of this survey have gained a better understanding of the poultry egg intake among residents in Kunming city and also suggested the development of more accurate egg intake guidelines as well as provided basic data for further nutrition monitoring and dietary exposure risk assessment via poultry egg intake in this area.

## Data availability statement

The original contributions presented in the study are included in the article/Supplementary material, further inquiries can be directed to the corresponding author.

## Ethics statement

The studies involving humans were approved by the Ethics Committee of Kunming Medical University. The studies were conducted in accordance with the local legislation and institutional requirements. Written informed consent for participation in this study was provided by the participants' legal guardians/next of kin.

## Author contributions

RW: Data curation, Investigation, Visualization, Methodology, Writing – review & editing, Writing – original draft. YW: Data curation, Investigation, Writing – review & editing. CZ: Data curation, Investigation, Writing – review & editing. CL: Data curation, Investigation, Writing – review & editing. XX: Data curation, Investigation, Writing – review & editing. XG: Data curation, Investigation, Writing – review & editing. JF: Conceptualization, Data curation, Methodology, Supervision, Writing – review & editing, Writing – original draft.
